# Daily Quality Assurance Efficiency Evaluation Using SunCHECK Machine and Machine Performance Check

**DOI:** 10.7759/cureus.35695

**Published:** 2023-03-02

**Authors:** Cassandra Stambaugh, Jessica Yancey, Utkarsh Shukla, Christopher Melhus, Nathaniel Stambaugh

**Affiliations:** 1 Radiation Oncology, Tufts Medical Center, Boston, USA; 2 Mathematics, Dexter Southfield School, Brookline, USA

**Keywords:** methods for quality assurance, quality assurance & improvement, daily quality assurance, external beam radiation therapy, efficiency, quality assurance

## Abstract

Purpose

To investigate time efficiency, applicability, and accuracy of using a web-based, independent quality assurance (QA) platform and vendor-dependent based system check for daily linear accelerator (LINAC) QA.

Methods

Time needed to perform daily QA on a single (n=1) LINAC was collected for three months. Task Group report 142 (TG-142) compliant daily QA included dosimetry checks (four photon, four electron beams); imaging checks (planar kilovolt (kV) & megavolt (MV), kV cone-beam computed tomography (CBCT)); and mechanical and safety checks using SunCHECK Machine (SCM) (Sun Nuclear Inc., Melbourne, FL, USA). Additionally, Machine Performance Check (MPC) (Varian Medical Systems, Inc., Palo Alto, CA, USA) was performed for all energies. Four trained radiation therapists performed daily QA on both platforms. Data were collected to identify the time required to complete both SCM and MPC. Additionally, the two platforms were evaluated on usability and features. Output results were compared to our monthly standard to assess accuracy.

Results

On average, SCM took 22 minutes with a standard deviation of six minutes and MPC took 15 minutes with a standard deviation of three minutes. MPC output results were impacted due to the beam output being coupled to the beam profile changes. As a result, the two systems on average disagreed by -1.41% after three months despite being baselined at the same time point and output agreeing well initially (average difference of -0.1% across all energies). While there was overlap in the tests performed, SCM tests were more relevant to TG-142 while MPC tests were beneficial to machine service and, with a clear understanding of the limitations of the system, found suitable as a secondary backup to SCM for daily output verification.

Conclusions* *

This work demonstrates that a comprehensive TG-142 daily QA can be designed using SCM and MPC can be added as a beneficial tool and backup for output verification while still maintaining an efficient daily QA process.

## Introduction

The increase in treatment complexity, personalized treatment plans, and combined treatment techniques in radiation oncology clinics increases the demands and importance of routine quality assurance (QA). With many needs to address, it is necessary to simplify processes and increase efficiency while maintaining the highest level of quality and safety. While the publication of Task Group 100 report (TG-100) [1] generically and Medial Physics Practice Guideline 8a (MPPG 8a) [2] specifically address methodologies to simplify QA to focus on critical safety processes, there have been very few publications looking at the efficiency of different QA platforms [3]. Additionally, when vendors supply integrated self-check tools to verify critical functions, clinics question how those tests could be used to increase the efficiency of QA tests and whether this practice is appropriate [4].

In order to maintain high levels of quality and safety, efficiency should only be one factor in determining the appropriate tool for QA. QA platforms should also be assessed on accuracy, applicability, and adaptability to the needs of a clinic and pertinent recommendations and guidelines, such as Task Group 142 report (TG-142) [5]. Additional features such as usability, accessibility, and data analysis/trending are also considerations when choosing a QA platform, and these features impact efficiency. This work evaluates the efficiency of using SunCHECK Machine (SCM) (Sun Nuclear Inc., Melbourne, FL, USA) and Machine Performance Check (MPC) (Varian Medical Systems, Inc., Palo Alto, CA, USA) for daily QA (DQA), and reviews platform-specific features that ultimately impact overall QA efficiency.

## Materials and methods

SunCHECK machine

SCM is a browser-based QA platform that collects, detects, analyzes, and stores QA data. The platform comes with built-in templates for TG-142 recommended DQA tests, and the templates can be customized for the specific needs of the clinic. It can be utilized for all machine QA, though this work focuses on DQA. It utilizes the Daily QA 3 (DQA3) (Sun Nuclear Inc.), fully automating beam constancy checks. The DQA3 contains 12 diodes for light-radiation field coincidence, four ion chambers for photon energy checks, four ion chambers for electron energy checks, and five ion chambers for flatness and symmetry. Upon initialization, the DQA3 collects all available beam parameters, and results are automatically analyzed in SCM. It is well described and validated by Binny et al. [6]. Additionally, SCM utilizes the IC Profiler (ICP) (Sun Nuclear Inc.) for monthly QA (MQA). The ICP comprises 251 ion chambers with 5 mm spacing along the X and Y axis and 7.07 mm spacing along the diagonals.

DQA at our institute consists of seven test groups, including 103 data elements collected. These tests included safety, mechanical, imaging, wedge, multi-leaf collimator (MLC), and dosimetry tests (6, 10, 10 flattening filter-free (FFF) and 18 megavolts (MV) & 6, 9, 12, and 18 megaelectronvolts (MeV)) and surface guidance tests. Dosimetry tests (68 of 103), including output, flatness & symmetry, energy, and field size, were collected using the DQA3. All other tests were performed manually and verified either with pre-built plans such as the qualitative MLC test and wedge run-out or physically such as checking lasers, door interlocks, etc. or with the onboard imaging systems.

Machine Performance Check

Varian developed MPC to provide an integrated self-check tool to verify the critical functions of the system [7]; in this case, the TrueBeam Linear Accelerator (LINAC) (Varian Medical Systems, Inc., Palo Alto, CA). MPC uses an IsoCal Phantom, a phantom with embedded ball bearings, which is well described by Gao et al. [8]. MPC is fully automated using the onboard kilovolt (kV) and MV imaging systems and is dependent on the IsoCal calibration [9], performed monthly in our institution.

Overall, 38 data elements were analyzed in nine MPC tests for our clinic. MPC tests included geometry checks for isocenter, collimator, gantry, and couch and beam checks of output, uniformity, and center shift for five photon energies (2.5, 6, 10, 10 FFF, and 18 MV) as well as output and uniformity for four electron energies (6, 9, 12 and 18 MeV). Further descriptions of the MPC geometry and beam output tests are outlined in Clivio et al. [10].

Time and platform analysis

Time Analysis

Our radiation therapists were informed that we would assess the time required to perform DQA on a single (n=1) LINAC for five months. Four experienced radiation therapists rotated through performing DQA, including MPC and SCM, during this time. Instructions were provided to perform all tests with no breaks, record any issues or reasons for delays, and repeat failed tests immediately to assess if it was a real (machine) or system (platform software) failure. Since this was a change in typical practice, the first two months were a “break-in” period with frequent check-ins. After this period, time to perform SCM and MPC was collected without reminders or modifications to the DQA process. All data is presented, but time data during the break-in period was separately analyzed from data after the official start of data collection.

SCM automatically timestamps all individual test set groups when performed and when the DQA is completed. Time for DQA on SCM was recorded from the start of setup (an indicator added to SCM) to the time of QA completion. MPC automatically timestamps all tests in the log files. Time for MPC was recorded between the first and last time stamp (start of setup to completion of last test).

Basic statistics for the time data were performed in Microsoft Excel (2013) (Redmond, WA, USA). Additionally, statistical process control [11] was performed two months before (pre) and three months after (post) the official data collection start to identify outliers in the time data. Any time data outside of the warning or control limits were investigated.

Platform Analysis

**Figure 1:**
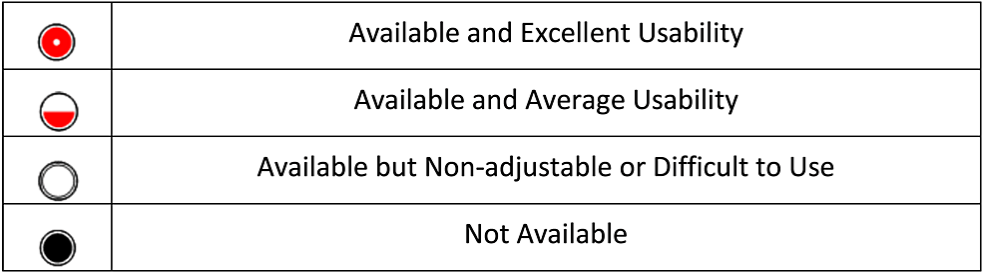
Ranking metrics utilized to analyze MPC and SCM on basic functionality and features MPC: Machine Performance Check, SCM: SunCHECK Machine

MPC and SCM were compared to the recommendations of TG-142 [5]. Additionally, each platform was ranked on ease of use, customizability, accessibility/notification, and trending/data management. The ranking metrics used were based on feature availability and level of usability per author consensus. They are presented in Figure 1.

Before the five months of data collection, a full annual calibration was performed, and baselines for all devices were taken or confirmed valid. No new processes were introduced for beam constancy checks during data collection.

Output constancy was measured daily using MPC and DQA3 in SCM. Additionally, measurements were taken weekly using the ICP. Output percent differences from baseline were calculated for each system and evaluated over time. Additionally, SCM was directly compared to MPC. Both systems were compared to the ICP output constancy results.

Modifications were introduced to the output and beam profile by stacking varying thicknesses of high-efficiency solid water (Sun Nuclear, Inc.) on the DQA3 for SCM and Electronic Portal Imaging Device (EPID) for MPC to assess the response of SCM and MPC under the influence of modified output. A similar process was previously described by Li et al. [12]. The thickness used ranged from 0.2 to 4 cm. Matlab (MathWorks, Natick, MA, USA) was used to obtain beam profiles for MPC and obtain central pixel intensity values. Central pixel intensity values were normalized to the maximum pixel intensity and compared to the normalized DQA3 and MPC output results as a function of solid water thickness in Excel. Additionally, the daily flatness and symmetry results for SCM and beam uniformity for MPC were evaluated over the measurement period.

## Results

Time analysis

**Figure 2:**
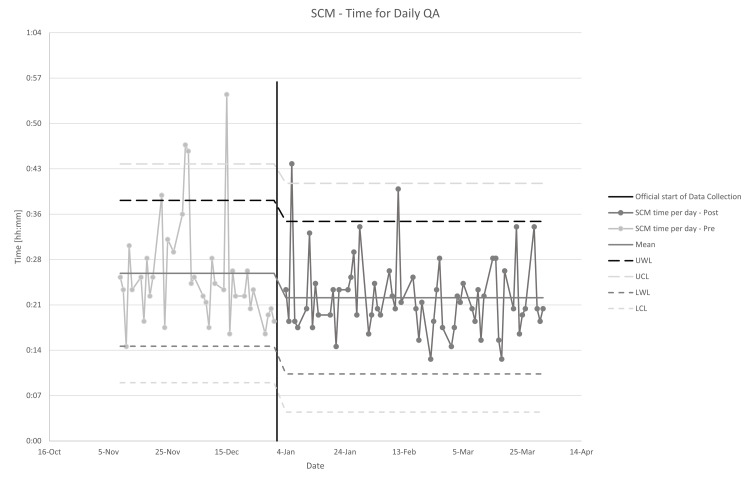
Time to perform daily SCM QA with warning and control limits from SPC analysis. Data is split at the point where the “break-in” period ended. SCM: SunCHECK Machine, QA: quality assurance, SPC: statistical process control, UWL: Upper Warning Limit, UCL: Upper Control Limit, LWL: Lower Warning Limit, LCL: Lower Control Limit

On average, SCM took 22 minutes (min 13, max 44, median 21) to perform with a standard deviation of six minutes. All of the times exceeding the upper warning level (38 minutes pre, 34 minutes post) in Figure 2 corresponded to days where repeat testing was needed due to software or machine failures preventing timely completion of the QA. These did not correspond with an individual user. The radiation therapist indicated that tests needed to be repeated due to a failure three times, a rate of 5%. As can be seen in Figure 2, there was a statistically significant decrease in average time (26 to 22 minutes, p=0.014, one-tailed t-test for comparison of means) and an increase in the standard deviation that did not reach significance (five to six minutes, p=0.334, Fligner-Killeen test for comparison of variances) after the initial break-in period.

**Figure 3:**
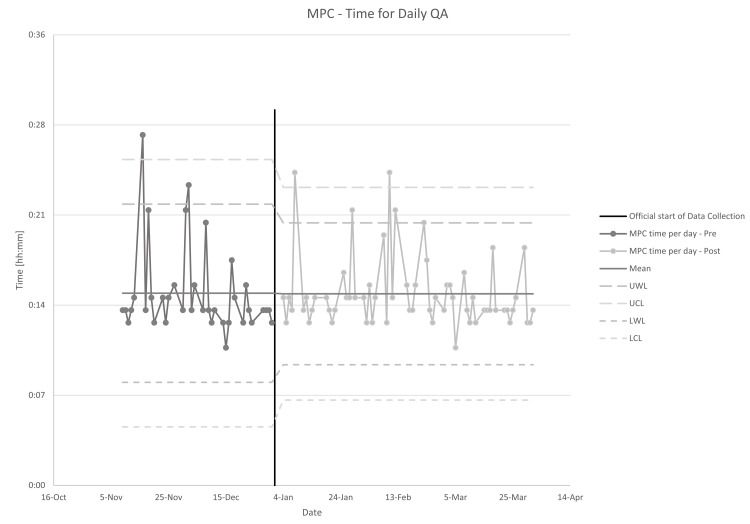
Time to perform daily MPC self-test with warning and control limits from SPC analysis. Data is split at the point where the “break-in” period ended. MPC: Machine Performance Check, SPC: statistical process control, UWL: Upper Warning Limit, UCL: Upper Control Limit, LWL: Lower Warning Limit, LCL: Lower Control Limit

On average, MPC took 15 minutes (min 11, max 25, median 14) to perform with a standard deviation of three minutes. The days exceeding the upper warning limit (UWL) in Figure 3 (22 min pre and 20 min post break-in period) mostly corresponded to days where repeated testing was needed. This was due to either phantom misalignment or results not recording in the MPC software. There was one instance where there was a delay between the end of one test and the start of the next, but the cause is unknown. These instances did not correspond with an individual user or test. Tests needed to be repeated due to failure eight times, a rate of 13% of the time. As can be seen in Figure 3, there was an insignificant decrease in average time (15.4 to 15.3 minutes) and a reduction in standard deviation (3.5 to 2.8 minutes) after the initial break-in period. These differences were not statistically significant (p=0.939, one-tailed t-test, p=0.750, Fligner-Killeen test) nor clinically significant.

Platform analysis

Each platform collects and records many machine parameters. Table 1 indicates which TG-142 recommendations are evaluated, where an “X” indicates that the platform can assess that parameter, and “X/-“ indicates that it can partially assess a parameter. MPC reports on beam output constancy, but since it is not designed as a platform for DQA, it does not collect data on mechanical or safety tests. While some of these checks could be assessed while MPC is being performed, there is no way to record results on the MPC platform. However, SCM can record the result of standard and custom mechanical and safety tests. The dosimetry measurements and analyses are automated with DQA3, while the remaining test results can be manually entered into the system.

**Table 1:** Platform analysis on the applicability to the recommendations of TG-142 for daily QA MPC: Machine Performance Check, SCM: SunCHECK Machine, TG-142: Task Group report 142, kV: kilovolts, MV: megavolts, EPID: Electronic Portal Imaging Device, EDW: Enhanced Dynamic Wedge

MPC	TG-142	SCM
Dosimetry		
X	X-Ray Output Constancy	X
X	Electron Output Constancy	X
Mechanical		
	Laser Localization	X
	Distance Indicator at Iso	X
	Collimator Size Indicator	X
Safety		
	Door Interlock	X
	Door Closing Safety	X
	Audiovisual Monitor(s)	X
	Stereotactic Interlock	X
	Radiation area monitor	X
	Beam on Indicator	X
Planar kV and MV (EPID) Imaging		
	Collision Interlocks	X
X/-	Positioning/Repositioning	X
X	Imaging and treatment coordinate coincidence	X
Cone-beam CT		
	Collision Interlocks	X
X/-	Positioning/Repositioning	X
	Imaging and treatment coordinate coincidence	X
EDW		
	Morning check-out run for one angle	X

MPC reports on the imaging units' positioning accuracy and the imaging and treatment isocenter coincidence for all imaging modalities. However, it does not evaluate repositioning or collision interlocks. It also does not assess the enhanced dynamic wedge (EDW). SCM does allow for data collection for these tests. They are part of the TG-142 template, but the results must be entered manually.

**Figure 4:**
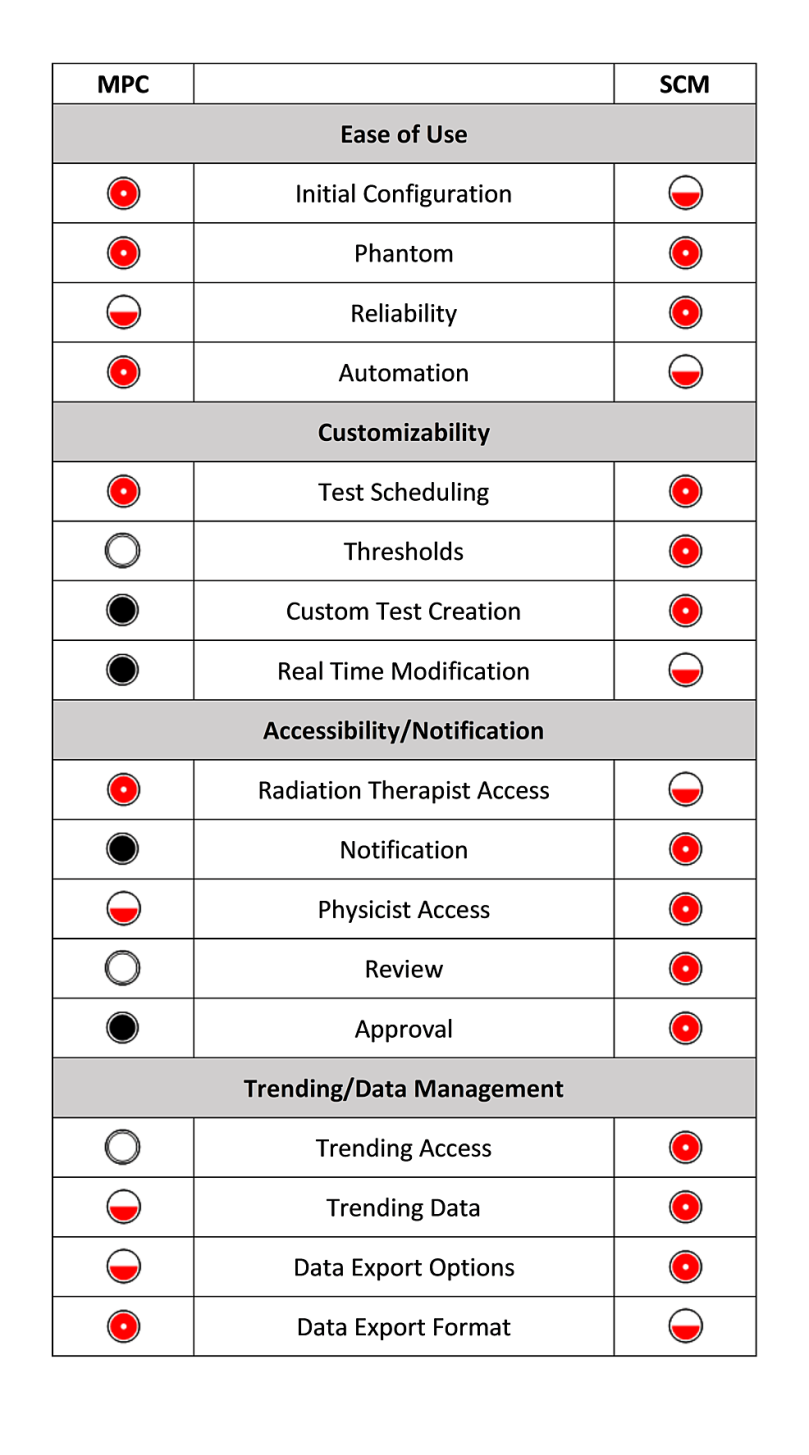
Ranking metric results for MPC and SCM on basic functionality and features. MPC: Machine Performance Check, SCM: SunCHECK Machine

Feature evaluation of each platform is presented in Figure 4. Both platforms are easy to use. Besides taking initial baselines for MPC, there is no additional setup. SCM has pre-built templates for DQA in compliance with TG-142. However, many baselines are needed, and if customization is wanted, additional time and iteration are required. For both platforms, each energy needs to be initiated for testing. More human interaction is needed for SCM as more custom tasks are added. The phantoms for both platforms are easy to set up and use. MPC required more repeat tests due to results not capturing. SCM auto saves results resulting in infrequent data loss.

Imaging tests for SCM use the Quasar Penta-Guide phantom (Modus Medical Devices, London, Canada). Cones are needed for electron dosimetry checks in SCM, while for MPC, they are not. Therefore, more trips into the room are required to complete DQA for SCM.

Both platforms can schedule tests, and individual tests can be scheduled according to the day of the week. One nuance is that radiation therapist accounts in SCM do not show tests as available if they are not scheduled. Therefore, the SCM schedule was modified to allow availability on Saturdays and Sundays in case of emergency treatments. Both platforms will propagate tests until completed, so if tests are not performed on the weekend or on a holiday, the QA is simply available on the next business day.

SCM is customizable to fit any DQA needs and allows for threshold adjustment if different thresholds are needed. MPC has thresholds, but they are not changeable nor are any of the tests.

SCM requires a login to a web application, which adds a step for radiation therapists, but does provide email notifications if desired. The platform displays results on a single web page and has a straightforward approval process. Since it is a web application, any networked computer can access the software. This, with the automatic notification of failed tests, was highly beneficial.

MPC is easier to access on the LINAC console, but there is no notification of failed tests to the physics group. There is an offline mode of MPC, which requires separate licensing and therefore not available on every workstation. Reviewing the individual test group results is non-intuitive, and there is no approval option to mark tests reviewed.

The multiple trending options in SCM (from the individual test or from the machine trending page) give the users flexibility in evaluating the data. The ability to see comments on the trend line is beneficial so that notes on outliers can be easily accessed. The trending in MPC is not as user-friendly, requiring more clicks to access.

**Figure 5:**
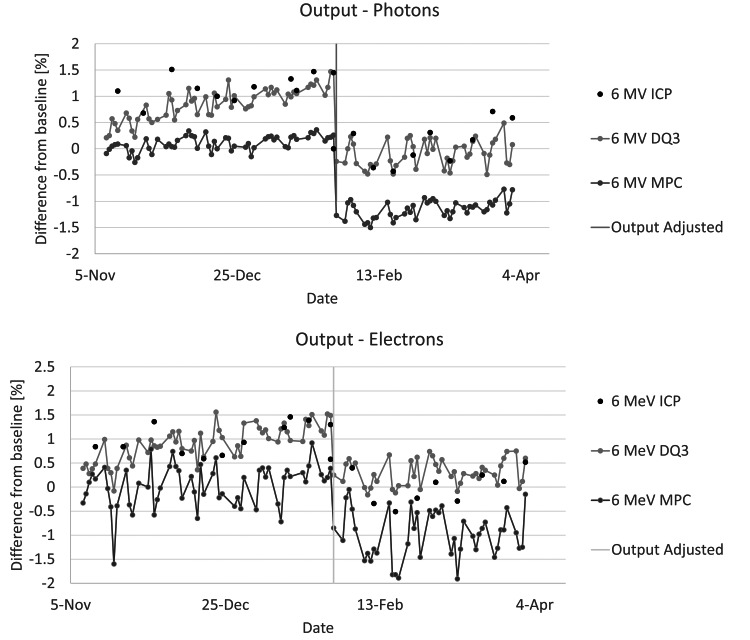
Output percent difference from baseline for ICP, DQA3, and MPC for different energies during our data collection period (6 MV & 6 MeV). ICP: IC Profiler, DQA3: Daily QA 3, MPC: Machine Performance Check, MV: Megavolts, MeV: Megaelectronvolts

We saw a slow upward trend in output over the first three months of data collection, which can be seen in Figure 5, represented by the 6 MV and 6 MeV data. While MPC and SCM directionally trended together, MPC did not demonstrate the same magnitude change as SCM. This was true across all energies. The output was adjusted at three months due to clinical need. MPC and SCM decreased by the same amount (Figure 5), at which point the measured output remained relatively constant for the remainder of the collection period. While the two systems were baselined and agreed well (average difference of -0.1% across all energies) initially, the two systems disagreed by -1.41% by three months. Differences in individual energies are shown in Table 2.

**Table 2:** Maximal and minimal output differences between MPC and SCM per energy MPC: Machine Performance Check, SCM: SunCHECK Machine, FFF: Flattening Filter-Free, MV: Megavolts, MeV: Megaelectronvolts

	6 MV	10 MV	18 MV	10 FFF	6 MeV	9 MeV	12 MeV	18 MeV
Max % Diff	-0.26	-0.38	0.07	0.03	-0.11	-0.33	-0.32	0.32
Min % Diff	-1.46	-1.65	-1.00	-1.19	-1.93	-1.65	-1.64	-0.81

Comparing each system to our monthly standard, we found good agreement (<0.5%) between our daily output constancy checks with SCM with the DQA3 and the ICP. MPC showed a systematic 0.5-1% difference between the ICP results for same-day measurements over all energies.

**Figure 6:**
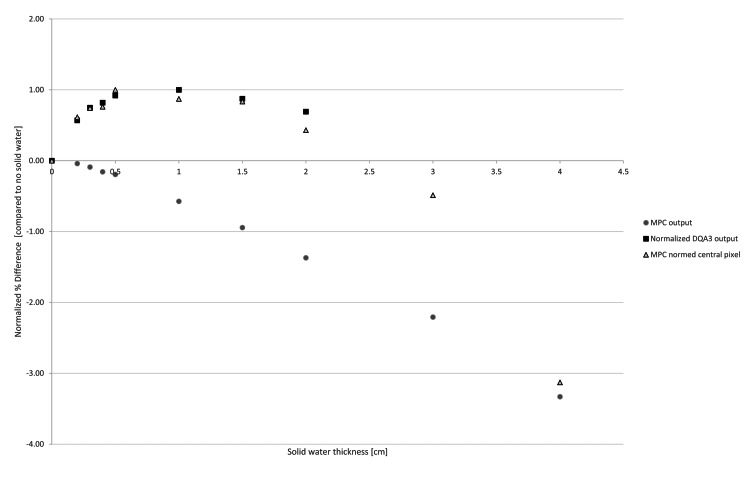
Changes in percent output difference per solid water thickness for MPC output, normalized DQA3 output and normalized central pixel MPC. MPC: Machine Performance Check, DQA3: Daily QA 3

**Figure 7:**
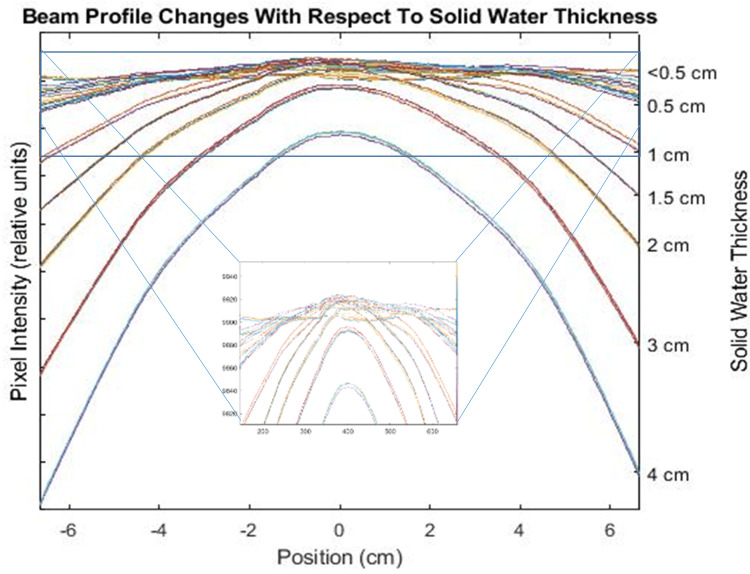
Beam profile changes with respect to solid water thickness placed on the EPID for MPC measurements; inset demonstrating the field changes with small beam modifications (<0.7 cm solid water) EPID: Electronic Portal Imaging Device, MPC: Machine Performance Check

Artificially creating changes in the beam profile and output demonstrated a similar phenomenon to what was seen in the output measurement results, where the reported MPC output measurement results did not align with DQA3 results. Figure 6 shows how the reported output for MPC does not trend with the reported output of the DQA3. However, the central pixel intensity normalized difference from baseline value trends well with the DQA3 results, demonstrating that MPC can report output constancy consistent with independent QA systems. However, MPC utilizes a 13.3 x 13.3 cm field to evaluate output, averaging the percentage of change over that field [6]. Figure 7 shows how the beam profile changed as the solid water was added over the 13.3 x 13.3 cm field that MPC analyzes for beam output results. While this intuitively makes sense, it demonstrates that even with small modifications to the beam (inset focusing on beam changes with <0.7 cm solid water), averaging over this field significantly impacts output results.

We reviewed our system's flatness and symmetry results when MPC diverged from DQA3 and found that all energies drifted in the beam profiles from baseline. 18 MeV demonstrated a minor change in flatness and symmetry during this period and correspondingly showed the least divergence between MPC and SCM. Therefore, MPC was underreporting the output results during this period, and at no point did MPC fail on uniformity. This could be rectified by reporting based on central pixel intensity but may impact efficiency.

## Discussion

Our institution's goal for DQA efficiency goal is less than 30 minutes per linear accelerator. Ideally, this would incorporate all necessary TG-142 tests as well as MPC for service records. This work demonstrates that TG-142-compliant DQA can be performed utilizing the SunCHECK platform and, even with additional dosimetry tests and clinic-specific checks, remain under 30 minutes. The time to perform MPC adds substantial time, pushing total DQA time out of our clinic's desired 30-minute time allowance.

In addition to the increased time, we found that for the majority of the MPC failures observed there was little information provided regarding the observed problem. Often the result simply did not record when there was an issue. Therefore, the user could only repeat the test or contact the vendor for guidance, which added additional time. Discrepancies in results between the two systems also caused confusion for the radiation therapists on whether there was an issue that would prevent treatment. Since evaluation of MPC results is limited to the treatment machine or a handful of dedicated workstations, any investigation of observed failures required notification from a therapist, opposed to SCM where any failure of a test automatically sent an email notification, increasing the burden on the staff.

In order to maintain a 30-minute allowance for DQA, the variation in QA time would need to be condensed, one platform could be used for the tests that overlap in both systems, or the number of tests would need to be reduced. Since the SCM platform does require more user interaction, more variation was seen overall and a learning curve was observed with the SCM system. Additional training or rearrangement of testing order could reduce the time spent performing the SCM tests. It was demonstrated that under ideal conditions, DQA for SCM could be performed in 13 minutes, leaving ample time for MPC checks.

The dosimetry tests are the area of greatest overlap between the two systems, and the natural place to look to help reduce time. However, we found across all energies, both photon and electron, MPC demonstrated an under-response to drift in output, an effect that could mask trending and delay. MPC has primarily been tested in two capacities in the literature: direct comparison to independent detectors and sensitivity to introduced errors. Four prior groups [12-15] have tested the accuracy of MPC on Varian’s TrueBeam system. While direct comparison demonstrates that beam output initially agrees between MPC and the DQA3, drift can be observed. There have been various explanations and suggestions as a result. Barnes et al. [13] found a drift in responses from MPC compared to the DQA3 with divergence over five months. It recommended regular inter-comparison checks between MPC to an ion chamber. Binny et al. [14] recommended calibration assessment of MPC every three months to account for inherent drifts in output with MPC. Pearson et al. [15] reported on three years of MPC data from eight machines, finding MPC output compared to ion chamber measurements averaged a difference of 0.2% but varied up to 2.9%. Li et al. [12] concluded MPC could detect changes in beam output and uniformity with sufficient accuracy/precision for DQA but did not report on MPC output results over time.

The drift in response seen in this work is consistent with Barnes et al. [13], however, the reasoning was not clearly explained in that work. Since MPC reports output based on the pixel intensity results of the central (13.3 cm)^2^, any changes in beam profile will influence the reported output. As seen in Figure 7, volume averaging is occurring for MPC that is not seen in the DQA3 or ICP results, as those use only central detectors to obtain output results and, therefore, are independent of beam profile changes.

As suggested by others [14], without frequent re-baselining of the MPC system, that system cannot be relied on for an independent check of output over time. The frequency of re-baselining and/or the tolerance in the difference between the two systems need to be considered when evaluating the efficiency of the program. The time savings gained by using MPC for dosimetry results may not be worth the amount of time spent on the additional tests to maintain the system. Additionally, the MPC dosimetry checks may not be considered an independent system, so it may not be a viable option based on institutional priorities. As such, our clinic views a well-maintained MPC system as a convenient and available secondary backup for output verification as opposed to a primary tool.

The number of checks performed by the combination of the two systems well exceeds the minimum requirements for DQA per TG-142 and MPPG 8a. The MPC geometry checks are the least pertinent to TG-142 and add a significant time to MPC checks. If MPC is desired, reducing the frequency of the geometry tests would result in a more efficient process over the course of the week while still maintaining a record of checks for service.

## Conclusions

Both platforms analyzed in this work proved to be easy to use. The use of SCM allows all relevant DQA data to be collected and stored in one platform while the use of MPC provides pertinent data for service engineers and a secondary dosimetry backup if maintained properly. While sufficient DQA could be performed using SCM in an idealized 30-minute time allowance, the addition of MPC checks as part of a daily routine would require additional work and consideration to reduce the overall DQA time. Efficiency evaluations, such as this one, allow adequate staffing and time allowances and reduce wasted time and resources - a necessary component of contemporary practice in an increasingly resource-constrained environment.
